# Dataset for PCBs and OCPs in intertidal sediment and surface-mixed layer water from Lagos lagoon, southwest coast of Nigeria

**DOI:** 10.1016/j.dib.2023.109645

**Published:** 2023-10-07

**Authors:** Nsikak U. Benson, John P. Unyimadu, Imokhai T. Tenebe

**Affiliations:** a Department of Chemical Sciences, Topfaith University, Mkpatak, Nigeria; b Institut des Sciences Analytiques, Universite ´ Claude Bernard Lyon 1, 5 rue de la Doua, F-69100 Villeurbanne, Lyon, France; c Department of Physical and Chemical Oceanography, Nigerian Institute for Oceanography and Marine Research, Lagos, Nigeria; d Mineta Transportation Institute, San Jose State University, USA

**Keywords:** Organic micropollutant occurrence, Sediment contamination, Polychlorinated biphenyls, Organochlorinated pesticides, Surface water pollution

## Abstract

Sediment quality and the integrity of coastal aquatic ecosystems are deteriorating, particularly in regions with unregulated discharges of chemical pollutants into the environment. Although organochlorine pesticides (OCPs) and polychlorinated biphenyls (PCBs) have been banned in recent decades for their adverse effects on the environment and human and animal health, these organic micropollutants are frequently detected in many aquatic systems. This dataset reports baseline concentrations of 27 PCBs and 20 OCPs analysed in surface-mixed layer water and sediment samples collected from designated sampling locations in the Lagos lagoon, off the Gulf of Guinea, Nigeria. The EPA Method 3570 (SW-846) and EPA Method 3510C (SW-846) with slight modifications were used for sediment and water extraction/cleanup procedures, respectively. Data were acquired using gas chromatography (GC) Agillent 6890 and 5890 equipped with electron capture detector (ECD). Data are reported in ng/L and µg/kg for concentrations of OCPs and PCBs in surface-mixed layer water and sediment samples, respectively. The interpretation of this dataset is fully discussed in the related research articles by Benson et al. (2023) and Unyimadu and Benson (2023).

Specifications Table

**Table:** 

Subject	Environmental chemistry, Pollution
Specific subject area	Sediments and estuarine water contamination and ecological risk assessment.
Data format	Raw Analysed
Type of data	Table Graph Figure
Data collection	Data were acquired using gas chromatography (GC) combined with electron capture detection (ECD)Instruments: • Agilent GC-ECD Model 6890 with ^63^Ni ECD equipped with a Rtx 5 fused capillary column (30 m x 0.25 mm inner diameter x 0.5 µm). • A Hewlett Packard Model 5890 Gas Chromatograph Series 11 coupled with an electron capture detector. The temperature conditions of the oven column were as follows: initial 180°C (for 1 min), increased by 10°C/min to 225°C and ramped to 250°C at 10°C/min (held for 10 min). The temperature of injector and detector was maintained at 250°C and 325°C, respectively. Purified N_2_ gas (99.99%) served as the carrier gas and was maintained at a flow rate of 1.0 mL/min.
Data source location	Nigerian Institute for Oceanography and Marine Research, Lagos, Nigeria.
Data accessibility	https://data.mendeley.com/datasets/v6fgnbjbxk/1 (Raw)
Related research article	N.U. Benson, J. P. Unyimadu, I.T. Tenebe, Distribution of organochlorine pesticides (OCPs) in surface-mixed layer water and intertidal sediments of Lagos lagoon, Gulf of Guinea. Regional Studies in Marine Science. (2023), 103187, https://doi.org/10.1016/j.rsma.2023.103187

## 1 Value of the Data


 

• Comprehensive baseline data on polychlorinated biphenyls (PCBs) and organochlorinated pesticides (OCPs) in the intertidal sediments and surface-mixed layer water of Lagos lagoon is presented.
• Sampling, analytical methods, and data described in this article will be helpful to researchers interested in organic micropollutants in aquatic environments, particularly individuals conducting systematic and meta-analysis reviews.
• Using these data, researchers can determine the primary sources of OCPs and PCBs, their environmental fate and toxicity.
• These data can be used in future environmental monitoring studies to determine the ecological and health risks posed by OCP and PCB exposures.
• The data could assist policymakers in selecting site-specific monitoring and remediation strategies to address contamination concerns.

## 2 Objective

The primary objective of this dataset is to provide baseline information on the pollution status of an important microtidal lagoon system using sediment and water samples obtained during a multi-location sampling survey. We investigated and determined the concentration and spatial distribution of PCBs and OCPs in sediment and surface-mixed layer water samples from Lagos lagoon, Nigeria [1], [2], [3]. Additionally, we estimated the potential ecological risk using sediment quality standards as well as the health risks associated with organism-level exposure to target organic micropollutants.

## 3 Data Description

The dataset presented here include raw data, which can be accessed at the Mendeley Data online repository. The raw data, which are presented as table in .xlsx, format, can be found at https://data.mendeley.com/datasets/v6fgnbjbxk/1 [1]. The data present a baseline concentrations and distribution characteristics of 27 polychlorinated biphenyls (PCBs) (PCB-8, 18, 28, 44, 52, 60, 77, 81, 101, 105, 114, 118, 123, 126, 128, 138, 153, 156, 157, 167, 169, 170, 180, 185, 189, 195, and 206) and 20 organochlorinated pesticides (OCPs) (α-BHC, β-BHC, γ-BHC (lindane), σ-BHC, hepthachlor, heptachlor epoxide, α-chlordane, γ-chlordane, aldrin, endrin, dieldrin, endrin aldehyde, endrin ketone, endosufan 1, endosulfan 11, endosulfan sulfate, p,p′-DDE, p,p′-DDD, p,p′-DDT, methoxychlor) were analysed in surface-mixed layer water and sediments samples collected from designated sampling locations in the Lagos lagoonal ecosystem off the Gulf of Guinea (Southeast Atlantic). Table 1 provides comprehensive information about sampling sites, GPS coordinates, and descriptions of possible sources of anthropogenic contaminations. Table 2, Table 3, Table 4, Table 5, Table 6, Table 7, Table 8, Table 9, Table 10, Table 11, Table 12, Table 13, Table 14 show the raw concentrations of organic micropollutants detected in sediments and water samples analysed [2,3]. Similar studies have documented the concentration, fate and risk assessments of other major hydrophobic organic contaminants [4] and microplastic-sorbed micropollutants [5], [6], [7] in sediment samples of the Lagoon and coastal beaches in the region.

**Table 1:** Locations of sample collection and descriptions of potential contamination sources.

Sampling location	GPS details	Description
1. Ibese	N 06^o^ 32.116’ E 003^o^ 29.534’	Sand mining and dredging
2. Nichemtex	N 06^o^ 34.731’ E 003^o^ 28.557’	Industrial effluents
3. Ikorodu Port	N 06^o^ 36.032’ E 003^o^ 28.835’	Industrial effluents
4. Ogudu/Agboyi	N 06^o^ 32.940’ E 003^o^ 24.489’	Solid waste dumps public toilets and residential building. Also receives effluent rich waste from Ogba/Ikeja industrial estate and Ogun River.
5. Oworonshoki	N 06^o^ 31.665’ E 003^o^ 24.466’	Wooden/Brick residential buildings lining the coastline, solid waste dumps in several locations and sand mining.
6. Unilag Front	N 06^o^ 31.048’ E 003^o^ 24.473’	Sewage discharge from university and neighboring communities, coolants from electricity sub-station, coastal residence and public toilets
7. Makoko	N 06^o^ 29.635’ E 003^o^ 23.787’	Wooden residential buildings along the coast and direct toilets, fishing, boat transportation, saw mills and high boat transport density
8. Okobaba	N 06^o^ 28.714’ E 003^o^ 23.426’	Log transportation and marketing, saw milling and wood burning, wooden residential buildings, solid waste dumps, public toilets, high boat transport density, receives road side dirt from the third mainland bridge
9. Iddo	N 06^o^ 28.190’ E 003^o^ 23.064’	Public toilets, solid waste dump, direct defecation, auto mechanic workshop, sac chemical plastic washing, boat transport and an abandoned power plant. Saturated foul-smelling air.
10. Ijora	N 06^o^ 27.414’ E 003^o^ 22.279’	Abandoned National Electric Power Authority facility discharge of transformer oil and municipal waste
11. Apapa Port	N 06^o^ 26.430’ E 003^o^ 22.572’	Oil tanker jetties, municipal waste discharge from nearby on-shore residence. Water surface dirty, oil sleek.
12. NIOMR Jetty	N 06^o^ 25.148’ E 003^o^ 24.244’	Marine Debris
13. Atlas Cove	N 06^o^ 25.191’ E 003^o^ 23.519’	Accidental Petroleum discharge, dredging, oil, grease and lead combustion
14. Park View	N 06^o^ 27.337’ E 003^o^ 27.092’	Residential effluents accumulation
15. Queens Drive	N 06^o^ 27.040’ E 003^o^ 26.648’	Residential effluents accumulation
16. Moba	N 06^o^ 27.724’ E 003^o^ 28.269’	Sand mining and Dredging
17. Ikate	N 06^o^ 28.228’ E 003^o^ 28.988’	Sand mining and Dredging
18. Egbin	N 06^o^ 33.209’ E 003^o^ 36.196’	Thermal Pollution, elevated water temperature

**Table 2:** Concentration (µg/kg) of PCBs in sediment samples from the Lagos lagoon.

Congener	Sample Code																	
	SD1	SD 2	SD3	SD 4	SD 5	SD 6	SD 7	SD8	SD 9	SD10	SD11	SD12	SD13	SD14	SD15	SD16	SD17	SD18
PCB-8	22.63	7.36	30.26	47.68	17.82	20.46	7.32	7.31	8.20	178	123	112	86.59	26.34	251	125	110	98.60
PCB-18	9.70	4.52	3.90	55.60	49.70	70.46	9.70	8.20	6.45	287	165	150	124	75.61	250	99.67	105	88.69
PCB-28	26.67	25.31	22.43	94.12	125	153	163	155	140	470	222	140	334	122	628	240	136	220
PCB-44	14.33	14.19	10.37	112	102	129	33.66	44.20	33.20	1074	201	180	171	166	104	220	212	189
PCB-52	5.46	9.96	2.73	29.80	44.21	49.77	62.40	55.43	45.32	558	44.50	33.65	55.08	50.73	630	70.55	55.26	33.64
PCB-60	32.83	51.78	18.63	63.05	118	65.90	108	212	216	1409	33.56	44.68	49.10	42.19	842	54.99	44.89	56.38
PCB-77	8.00	20.56	10.46	52.51	10.42	83.70	24.76	12.78	14.56	20.23	60.22	58.21	78.70	94.15	32.18	66.39	54.21	73.25
PCB-81	33.60	60.85	27.60	61.22	81.34	52.66	34.83	23.89	24.32	213	112	98.22	229	35.24	367	112	110	121
PCB-101	2.63	23.99	1.81	8.28	112	12.80	6.13	9.00	8.20	69.20	61.33	65.33	40.64	223	213	56.90	39.55	44.65
PCB-105	17.10	17.52	14.03	203	65.23	88.86	161	153	144	128	121	120	334	105	28.91	88.56	56.55	90.67
PCB-114	9.33	9.14	5.27	78.90	99.04	159	81.83	66.45	55.67	168	111	98.50	151	7.90	74.29	91.24	43.80	32.33
PCB-118	0.82	2.31	1.41	18.91	14.61	9.47	3.56	1.24	3.54	41.63	26.55	12.60	13.34	22.29	27.72	88.36	23.89	23.56
PCB-123	3.83	2.34	1.75	17.78	28.95	55.03	30.83	24.21	23.14	16.30	33.43	23.89	55.08	11.56	90.23	56.90	43.22	21.44
PCB-126	0.87	3.86	7.16	7.32	10.19	22.73	4.20	1.20	0.90	71.00	55.32	40.32	68.26	33.59	11.52	16.58	45.99	36.90
PCB-128	ND	5.77	2.14	14.66	29.34	22.50	18.53	19.56	16.55	48.20	44.33	32.56	30.60	34.02	82.05	40.67	30.56	28.55
PCB-138	ND	9.47	3.40	5.76	46.12	23.33	14.32	12.55	12.90	178	98.45	112	279	9.17	153	123	131	90.56
PCB-153	ND	ND	ND	65.95	18.38	74.40	41.65	33.65	34.22	47.53	45.90	33.29	46.35	189	24.55	30.78	48.76	43.55
PCB-156	59.60	ND	30.33	121	54.57	114	96.93	88.32	83.21	45.47	23.66	32.33	120	41.69	61.08	42.66	33.99	40.56
PCB-157	ND	16.66	1.66	27.32	30.69	46.96	29.03	28.56	34.66	11.73	13.56	12.33	23.71	42.75	ND	51.98	12.75	44.28
PCB-167	1.41	1.11	11.67	ND	128	125	87.10	77.25	65.89	46.63	34.22	41.25	43.65	146	ND	55.64	23.66	32.66
PCB-169	2.84	2.89	1.24	147	18.32	69.30	45.80	33.58	54.88	216	28.55	42.21	46.68	33.35	144	34.66	46.12	30.56
PCB-170	1.19	1.16	1.69	54.43	75.92	59.10	64.50	44.78	43.21	194	78.33	55.33	89.66	95.04	115	56.33	29.45	77.59
PCB-180	2.55	1.87	1.62	74.67	174	102	87.50	60.23	55.32	63.36	45.32	41.28	54.72	55.74	49.86	41.89	55.32	36.89
PCB-185	1.28	2.62	0.12	75.79	90.88	39.33	21.03	18.45	34.23	129	38.56	28.56	73.24	71.46	84.79	50.33	49.56	67.55
PCB-189	0.66	0.79	0.94	18.68	15.95	24.83	36.53	22.58	22.56	28.33	41.22	32.66	39.00	21.22	17.62	23.56	21.67	33.29
PCB-195	8.13	6.86	9.57	1148	493	619	1047	987	872	876	129	132	1228	1230	159	123	100	89.56
PCB-206	8.00	8.68	9.67	211	284	91.57	249	200	196	170	33.23	23.88	169	25.28	333	44.68	67.38	51.00

ND = Not detected.

**Table 3:** Concentration (µg/kg) of hexacyclohexanes (HCHs), aldrin and dieldrin in sediment samples.

Sample Code	α-HCH	β-HCH	γ-HCH	δ-HCH	Aldrin	Dieldrin
SD1	10.06	19.51	17.98	28.21	64.90	32.07
SD2	0.93	2.54	1.86	3.18	7.53	2.51
SD3	8.19	0.01	34.00	28.40	45.19	16.75
SD4	7.03	11.99	30.80	20.38	70.10	6.86
SD5	30.47	32.43	24.71	45.89	99.20	37.63
SD6	17.82	40.13	55.51	22.64	59.94	18.18
SD7	22.71	17.65	8.23	24.01	52.55	7.13
SD8	12.02	37.26	21.29	32.93	45.72	6.66
SD9	13.55	23.31	22.64	20.18	53.38	32.77
SD10	30.30	46.02	50.65	14.22	902.3	68.43
SD11	3.03	3.63	3.86	4.73	16.85	8.69
SD12	23.71	23.61	18.81	34.93	106..9	31.04
SD13	13.42	30.30	9.36	27.84	67.33	19.58
SD14	12.69	36.80	12.05	31.24	68.53	ND
SD15	11.50	26.23	30.11	ND	54.60	12.32
SD16	30.47	32.43	24.71	45.89	99.20	37.63
SD17	11.16	14.99	12.85	23.41	77.59	ND
SD18	29.70	24.39	45.19	21.51	207.7	24.38

ND = Not detected.

**Table 4:** Concentration (µg/kg) of chlordanes in sediment samples.

Sample Code	γ-Chlordane	α-Chlordane	Heptaclor	Heptaclor epoxide	Methoxyclor
SD1	8.49	8.33	65.97	72.73	9.52
SD2	2.31	1.35	7.29	17.32	6.23
SD3	7.23	7.99	39.66	45.19	8.49
SD4	8.29	9.82	46.02	43.29	24.34
SD5	21.91	93.67	224.89	11.75	7.06
SD6	10.79	14.62	43.39	76.22	13.19
SD7	11.72	23.48	119.85	133.40	15.88
SD8	7.69	7.77	62.27	75.72	11.15
SD9	39.43	87.11	171.23	87.61	14.38
SD10	76.16	187.48	312.85	296.90	12.99
SD11	2.36	5.13	16.62	25.67	1.65
SD12	12.52	39.06	182.55	62.64	18.12
SD13	8.26	26.87	42.52	50.78	30.84
SD14	8.76	9.59	66.80	80.22	10.96
SD15	3.29	56.12	ND	43.11	13.14
SD16	21.91	93.77	224.84	11.76	7.06
SD17	8.92	29.27	94.97	69.13	13.79
SW18	14.42	53.31	143.59	128.44	17.55

ND = Not detected.

**Table 5:** Concentration (µg/kg) of endrin and metabolites in sediment samples.

Sample Code	Endrin	Endrin Aldehyde	Endrin Ketone
SD1	ND	ND	49.95
SD2	ND	ND	3.60
SD3	ND	ND	16.15
SD4	66.67	ND	16.28
SD5	6.46	11.72	25.07
SD6	22.28	62.00	14.95
SD7	26.27	52.54	51.48
SD8	7.09	50.98	16.95
SD9	3.73	21.84	19.71
SD10	ND	44.62	28.80
SD11	ND	13.69	2.01
SD12	14.39	21.35	100.17
SD13	ND	ND	9.19
SD14	ND	ND	35.70
SD15	ND	25.33	51.93
SD16	ND	11.72	25.07
SD17	ND	34.30	31.24
SD18	13.45	64.27	39.93

ND = Not detected.

**Table 6:** Concentration (µg/kg) of DDT and metabolites in sediment samples.

Sample Code	DDT	DDE	DDD
SD1	ND	40.33	405
SD2	ND	1.72	161
SD3	ND	ND	394
SD4	ND	ND	393
SD5	ND	6.93	411
SD6	ND	48.92	296
SD7	ND	18.25	342
SD8	ND	32.70	268
SD9	ND	5.66	132
SD10	ND	ND	461
SD11	ND	3.14	101
SD12	ND	26.87	356
SD13	ND	ND	441
SD14	ND	34.83	543
SD15	ND	ND	457
SD16	ND	6.93	411
SD17	ND	ND	243
SD18	ND	32.97	230

ND = Not detected.

**Table 7:** Concentration (µg/kg) of endosulfan and metabolites in sediment samples.

Sample Code	Endosulfan 1	Endosulfan 11	Endosufan Sulfate
SD1	5.56	ND	132.43
SD2	8.29	ND	30.10
SD3	35.56	ND	62.30
SD4	ND	ND	48.78
SD5	6.79	ND	86.26
SD6	8.99	ND	66.07
SD7	6.76	ND	81.72
SD8	13.62	ND	78.95
SD9	8.09	99.87	43.36
SD10	ND	ND	52.58
SD11	1.40	ND	8.89
SD12	8.03	ND	35.53
SD13	19.58	ND	41.86
SD14	6.76	ND	71.86
SD15	2.34	ND	4.50
SD16	6.79	ND	86.28
SD17	ND	ND	22.68
SD18	10.09	53.85	59.21

ND = Not detected.

**Table 8:** Concentration (ng/L) of hexacyclohexanes (HCHs), aldrin and dieldrin in water samples.

Sample Code	α-HCH	β-HCH	γ-HCH	δ-HCH	Aldrin	Dieldrin
SW1 (Ibese)	2.79	6.66	10.94	5.37	11.52	1.59
SW2(Nichemtex)	0.29	0.57	0.36	1.04	2.44	ND
SW3 (Ikorodu Port)	1.73	2.16	5.09	2.16	11.34	ND
SW4 (Agboyi/Ogudu)	2.08	3.26	4.02	5.70	8.62	17.25
SW5 (Oworonshoki)	0.82	1.07	2.60	1.34	3.25	1.98
SW6 (Unilag Front)	0.62	3.38	0.18	1.67	2.08	ND
SW7 (Makoko)	0.07	0.21	0.44	1.21	2.33	ND
SW8 (Okobaba)	1.29	2.98	3.62	4.71	9.41	ND
SW9 (Iddo)	0.98	1.90	2.22	2.31	1.56	ND
SW10 (Ijora)	0.46	0.43	1.15	0.82	2.96	1.23
SW11 (Apapa Port)	2.03	3.99	1.99	3.20	8.15	14.64
SW12 (NIOMR Jetty)	1.78	6.58	8.50	5.67	9.40	1.42
SW13 (Atlas Cove)	1.15	5.39	ND	5.37	4.98	19.15
SW14 (Park View)	0.21	1.03	0.64	1.03	1.13	2.92
SW15 (Queens Drive)	0.89	3.62	3.07	4.72	10.46	ND
SW16 (Moba)	0.77	3.48	3.42	3.33	5.24	2.10
SW17 (Ikate)	2.04	3.54	5.79	6.02	16.33	18.32
SW18 (Egbin)	2.20	3.33	3.21	5.60	8.65	7.15

ND = Not detected.

**Table 9:** Concentration (ng/L) of PCBs in water samples from the Lagos lagoon.

Congener	Sample Code																	
	SW1	SW2	SW3	SW4	SW5	SW6	SW7	SW8	SW9	SW10	SW11	SW12	SW13	SW14	SW15	SW16	SW17	SW18
PCB-8	21.8	15.5	12.9	4.00	8.60	5.70	0.60	3.50	24.50	2.40	8.70	26.0	13.10	16.9	5.60	8.80	7.00	3.30
PCB-18	7.10	5.80	10.60	6.30	10.7	10.8	1.40	2.80	6.40	8.60	2.20	3.60	4.00	1.10	9.00	5.50	4.40	5.60
PCB-28	12.1	8.00	8.60	17.0	16.9	19.3	9.60	4.30	11.2	17.70	7.30	7.50	6.60	7.60	20.00	15.60	9.00	8.80
PCB-44	6.90	6.00	6.60	4.70	13.70	13.70	12.40	11.50	6.00	20.3	5.80	5.60	5.30	5.90	34.40	8.80	7.50	6.00
PCB-52	2.50	4.10	4.60	5.60	7.50	6.80	2.70	3.30	7.20	6.30	3.80	3.60	3.40	4.70	7.50	6.60	5.50	4.60
PCB-60	2.00	26.70	8.00	5.60	6.80	9.90	3.30	8.50	3.80	15.80	11.00	2.20	1.80	2.00	16.00	10.00	5.60	8.60
PCB-77	7.10	10.90	7.20	1.30	19.1	7.60	2.10	2.60	14.00	7.20	7.20	9.20	8.80	3.70	8.80	5.30	6.60	5.80
PCB-81	21.1	23.30	18.20	9.50	9.60	4.10	4.90	17.20	25.5	3.00	20.30	15.10	14.60	16.00	4.00	3.00	4.00	3.50
PCB-101	3.50	2.50	1.30	21.60	1.90	4.10	4.90	17.2	2.55	16.80	4.40	1.90	0.14	0.14	17.8	8.80	4.80	3.50
PCB-105	4.20	5.70	4.90	2.00	9.10	7.20	2.70	5.50	5.60	8.30	5.10	3.60	4.90	3.90	7.80	4.50	6.60	4.50
PCB-114	6.80	5.50	6.00	2.70	17.30	12.90	3.30	8.10	5.90	17.1	6.40	6.00	0.70	6.40	19.00	8.80	9.00	7.50
PCB-118	8.00	2.30	2.20	2.60	1.90	1.20	0.10	2.10	2.50	2.10	3.60	2.10	2.40	1.80	3.10	2.00	1.00	2.00
PCB-123	4.80	12.30	2.50	5.90	8.10	2.00	0.40	3.30	3.30	8.30	2.70	1.90	2.40	2.60	9.00	7.80	6.60	4.50
PCB-126	5.80	1.90	27.20	0.80	2.00	2.10	0.40	1.70	6.10	1.40	2.40	5.70	2.90	5.60	3.00	4.00	2.50	2.20
PCB-128	13.30	3.90	5.60	3.20	1.70	3.10	0.03	0.50	11.6	3.30	2.70	1.80	2.70	1.60	2.80	2.20	2.90	3.40
PCB-138	32.50	3.20	2.90	0.90	10.90	1.10	0.40	25.80	3.10	10.7	23.2	5.50	20.70	6.40	9.90	2.10	6.70	3.40
PCB-153	3.00	25.40	50.60	29.60	9.20	7.80	1.00	5.00	ND	18.70	15.80	23.10	36.00	21.5	7.50	8.80	6.70	5.50
PCB-156	1.55	1.18	0.15	0.68	1.38	1.77	0.02	0.69	4.32	4.90	6.30	53.20	1.30	37.90	4.40	2.00	3.50	7.80
PCB-157	6.20	2.40	6.00	ND	9.90	9.80	5.50	2.10	12.2	5.60	1.90	9.90	15.60	20.00	4.40	2.00	3.50	7.80
PCB-167	9.80	4.00	2.20	1.90	17.70	21.0	1.80	4.80	19.3	21.5	6.70	2.60	2.80	3.70	11.40	5.60	6.60	7.50
PCB-169	3.70	4.00	2.20	1.90	17.70	21.0	1.80	4.80	19.3	0.60	4.70	1.30	7.80	2.40	7.60	8.80	6.70	6.60
PCB-170	3.50	8.00	3.60	9.60	7.80	2.80	0.50	1.10	3.10	16.40	3.80	4.50	7.80	3.30	4.50	6.70	8.80	6.70
PCB-180	12.00	11.90	11.70	13.10	7.50	12.90	0.80	11.70	4.40	19.00	5.70	8.40	15.50	15.90	9.00	6.70	4.50	2.20
PCB-185	2.50	2.10	2.20	21.10	3.10	13.10	5.90	6.10	9.90	10.40	2.40	5.40	5.10	5.20	10.00	8.80	5.60	8.90
PCB-189	10.90	7.60	7.80	2.10	6.70	8.10	0.70	14.7	2.90	2.40	8.10	7.30	6.90	7.00	2.00	6.50	3.40	6.80
PCB-195	40.8	38.40	39.80	144.2	127.8	43.20	0.03	5.30	12.7	177.2	34.50	24.70	71.00	37.5	143.3	34.50	26.70	20.00
PCB-206	7.00	71.30	71.60	11.00	4.00	26.10	2.00	117.8	41.7	14.90	84.5	62.10	60.30	58.60	26.70	34.40	45.6	37.80

ND = Not detected.

**Table 10:** Distribution and concentrations of polychlorinated biphenyl species groups (di-∑PCB – nona-∑PCB) at each sampling location.

	PCBs in sediment samples																	
Sum of homologues	SD1	SD2	SD3	SD4	SD5	SD6	SD7	SD8	SD9	SD10	SD11	SD12	SD13	SD14	SD15	SD16	SD17	SD18
∑di-PCB	22.63	7.36	30.26	47.68	17.82	20.46	7.32	7.31	8.20	178.00	123.00	112.00	86.59	26.34	251.00	125.00	110.00	98.60
∑tri-PCB	36.37	29.83	26.33	149.72	174.70	223.46	172.70	163.20	146.45	757.00	387.00	290.00	458.00	197.61	878.00	339.67	241.00	308.69
∑tetra-PCB	94.22	157.34	69.79	318.58	355.97	381.03	263.65	348.30	333.40	3274.23	451.28	414.76	582.88	388.31	1975.18	523.93	476.36	473.27
∑penta-PCB	34.58	59.16	31.43	334.19	330.02	347.89	287.55	255.10	235.45	494.13	408.63	360.64	662.32	403.34	445.67	398.54	253.00	249.55
∑Hexa-PCB	63.85	35.90	50.44	381.69	325.42	475.49	333.36	293.47	302.31	593.56	288.67	305.97	589.99	495.98	464.68	379.39	326.84	310.72
∑Hepta-PCB	5.68	6.44	4.37	223.57	356.75	225.26	209.56	146.04	155.32	414.69	203.43	157.83	256.62	243.46	267.27	172.11	156.00	215.32
∑Octa-PCB	8.13	6.86	9.57	1148.00	493.00	619.00	1047.00	987.00	872.00	876.00	129.00	132.00	1228.00	1230.00	159.00	123.00	100.00	89.56
∑Nona-PCB	8.00	8.68	9.67	211.00	284.00	91.57	249.00	200.00	196.00	170.00	33.23	23.88	169.00	25.28.00	333.00	44.68	67.38	51.00

	PCBs in water samples																	
	SW1	SW2	SW3	SW4	SW5	SW6	SW7	SW8	SW9	SW10	SW11	SW12	SW13	SW14	SW15	SW16	SW17	SW18

∑di-PCB	21.80	15.50	12.90	4.00	8.60	5.70	0.60	3.50	24.50	2.40	8.70	26.00	13.10	16.90	5.60	8.80	7.00	3.30
∑tri-PCB	19.20	13.80	19.20	23.30	27.60	30.10	11.00	7.10	17.60	26.30	9.50	11.10	10.60	8.70	29.00	21.10	13.40	14.40
∑tetra-PCB	39.60	71.00	44.60	26.70	56.70	42.10	25.40	43.10	56.50	52.60	48.10	35.70	33.90	32.30	70.70	33.70	29.20	28.50
∑penta-PCB	33.10	30.20	44.10	35.60	40.30	29.50	11.80	37.90	25.95	54.00	24.60	21.20	13.44	20.44	59.70	35.90	30.50	24.20
∑Hexa-PCB	70.05	44.08	69.65	38.18	68.48	65.57	10.55	43.69	69.82	65.30	61.30	97.40	86.90	93.50	48.00	31.50	36.60	42.00
∑Hepta-PCB	28.90	29.60	25.30	45.90	25.10	36.90	7.90	33.60	20.30	48.20	20.00	25.60	35.30	31.40	25.50	28.70	22.30	24.60
∑Octa-PCB	40.80	38.40	39.80	144.20	127.80	43.20	0.03	5.30	12.70	177.20	34.50	24.70	71.00	37.50	143.30	34.50	26.70	20.00
∑Nona-PCB	7.00	71.30	71.60	11.00	4.00	26.10	2.00	117.80	41.70	14.90	84.50	62.00	60.30	58.60	26.70	34.40	45.60	37.80

**Table 11:** Concentrations (ng/L) of chlordane pesticides in water samples.

Sample Code	γ-Chlordane	α-Chlordane	Heptachlor	Heptachlor Epoxide	Methoxyclor
SW1	1.53	4.08	9.74	10.71	3.12
SW2	0.36	0.25	1.39	5.53	1.08
SW3	0.99	0.99	5.12	6.65	ND
SW4	1.36	1.84	5.31	14.57	6.32
SW5	0.39	0.79	ND	5.15	2.25
SW6	0.47	0.45	0.61	4.06	ND
SW7	0.26	0.20	1.28	2.36	ND
SW8	1.05	0.69	ND	1.12	5.25
SW9	0.86	0.56	2.12	3.44	3.45
SW10	0.31	0.58	1.63	0.42	ND
SW11	1.41	1.23	4.36	8.67	ND
SW12	1.26	1.41	7.79	12.62	2.24
SW13	1.17	1.38	6.09	11.08	ND
SW14	ND	0.19	0.85	2.51	2.26
SW15	1.10	1.16	10.46	6.81	2.37
SW16	0.98	0.62	3.12	2.23	1.23
SW17	1.47	3.68	3.74	9.49	0.31
SW18	0.98	2.21	2.23	6.41	0.45

ND = Not detected.

**Table 12:** Concentration (ng/L) of endrin pesticides in water samples.

Sample Code	Endrin	Endrin Aldehyde	Endrin Ketone
SW1	2.22	10.68	1.75
SW2	0.46	3.28	2.05
SW3	ND	ND	10.56
SW4	0.89	8.76	2.47
SW5	25.70	1.25	0.27
SW6	ND	2.37	0.42
SW7	0.34	ND	3.54
SW8	ND	ND	2.53
SW9	ND	ND	2.34
SW10	2.41	3.52	0.91
SW11	0.06	ND	2.78
SW12	ND	9.86	15.14
SW13	ND	ND	1.63
SW14	ND	1.09	0.35
SW15	ND	4.14	1.82
SW16	0.56	2.12	1.21
SW17	ND	0.25	1.29
SW18	ND	0.21	1.12

ND = Not detected.

**Table 13:** Concentration (ng/L) of DDT and metabolites in water samples.

Sample Code	DDT	DDE	DDD
SW1	ND	2.64	47.79
SW2	ND	1.14	28.46
SW3	ND	4.84	106.3
SW4	ND	5.31	42.34
SW5	ND	0.73	13.37
SW6	ND	0.42	27.07
SW7	ND	ND	26.87
SW8	ND	ND	ND
SW9	ND	ND	ND
SW10	ND	0.24	17.96
SW11	ND	ND	63.21
SW12	ND	8.27	57.54
SW13	ND	ND	75.10
SW14	ND	ND	15.18
SW15	ND	ND	87.03
SW16	ND	ND	9.97
SW17	ND	ND	47.59
SW18	ND	ND	12.13

ND = Not detected.

**Table 14:** Concentration (ng/L) of Endosulfan and Metabolites in Water Samples.

Sample Code	Endosulfan 1	Endosulfan 11	Endosufan Sulfate
SW1	0.45	ND	ND
SW2	ND	ND	5.81
SW3	ND	ND	14.12
SW4	ND	ND	4.97
SW5	ND	ND	18.20
SW6	ND	ND	5.97
SW7	ND	ND	4.12
SW8	ND	ND	2.56
SW9	ND	ND	1.89
SW10	ND	ND	4.29
SW11	ND	ND	10.88
SW12	ND	ND	4.26
SW13	ND	ND	7.97
SW14	ND	ND	1.11
SW15	ND	ND	3.08
SW16	ND	ND	2.31
SW17	ND	ND	14.81
SW18	ND	ND	8.21

ND = Not detected.

## 4 Experimental Design, Materials and Methods

### 4.1 Surface-mixed layer water sampling

Water samples were collected during the sampling period using a Van Don water sampler. The Van Don sampler used was manually operated and have open tubes of known volume fitted with a closure mechanism at each end. The water sampler was constructed of stainless steel. The sampling device is lowered on a calibrated line to the specific sampling depth, the sample is taken and the top and bottom lids are closed. 500 mL amber glass bottles that were used for the collection of surface and bottom water samples were completely decontaminated by washing in detergent to remove any solid residues, soaked for 24 h in an acid bath (chromic acid wash; 100 mL of concentrated H_2_SO_4_ slowly and with constant stirring to a solution of 5 g of sodium dichromate in 5 mL of water). The bottles were rinsed with deionized distilled water twice and allowed to dry in an oven over night. The bottles were rinsed twice with hexane.

### 4.2 Intertidal sediment sampling

A total of 54 sediment samples were collected from the sampling locations. Motorised boat was used for grabbing at all the locations. The upper 15 cm of the surface sediments were sampled from the sampling sites with a Birge-Ekman sediment grabber in areas of low flow velocity (<0.30 m/s). Immediately the grab is lifted to the boat a stainless-steel scoop precleaned with acetone was used to collect about 100 g of the sediment sample into a precleaned 200 mL amber glass bottled. The sample was preserved in a cooler with ice blocks. Immediately on land the sampled were preserved in a deep freezer to avoid degradation. Samples were stored for the shortest possible time interval between sampling and extraction/cleanup.

### 4.3 Reagents

In order to minimize sample contamination only glass, metal and Teflon equipment was used in the laboratory. Equipment was cleaned by agitation in detergent containing water and rinsed with high purity acetone and n-hexane prior to the extraction procedure. All solvents analytical grade (hexane, acetone, dichloromethane, petroleum spirit, acetonitrile) were purchased from Merck, Germany, and distilled over 0.5 m packed column (reflux ratio approximately 1:25). The solvent purity was tested by gas chromatographic analyses. Anhydrous granulated sodium sulfate, and silica gel 100 - 200 mesh (Merck, Germany) which was needed for the analytical procedure were cleaned with pure n-hexane. The external and internal standard, were purchased from Restek, USA, and was constituted of 1000 µg/mL of the following organochlorine compounds, α-BHC, β-BHC, γ-BHC, δ-BHC, endrin, endrin aldehyde, endrin ketone, heptachlor, heptachlor epoxide, adrin, dieldrin, endosulfane 1, endosulfane 11, endosulfane sulfate, methoxychlor, α-chlordane, γ-chlordane, DDE, DDT and DDT. The PCB external standard consisting of 27 congeners was also purchased from Restek USA. The congeners were PCB-8, -18, -28, -31, -44, -52, -60, -77, -74, -81, -101, -105, -114, -118, -123, -126, -128, -138, -141, -153, -156, -157, -167, -169, -170, -180, -185, -189, -195, and PCB-206. Frequent execution of blank analyses confirmed continuously the absence of laboratory derived contamination at concentration levels above the limit of detection (LOD) and quantification (LOQ).

### 4.4 Sediment extraction

The procedures EPA 3570 with slight modification were used [8]. Approximately 5.0 grams of anhydrous sodium sulfate was added to a precleaned mortar and 5 grams of fresh wet sediment was added to the mortar and homogenized to a complete mixture. The mixture was carefully transferred to a pre-cleaned PTFE extraction tube which has a PTFE screw cap. 5 to 10 pre-cleaned glass beads were added. 25 mL of a mixture of acetone and petroleum spirit (1:1) was added to the PTFE extraction tube; the extraction tube was tightly capped and allowed to stand for minimum of 20 minutes. This allows complete permeation of solvent to the matrix. 20 μg/L of the internal standard decaflourobiphenyl in iso-octane directly added to the sediment and sodium sulphate mixture. The tube was shaken vigorously until the slurry is free-flowing. Any chunks were broken with the metal spatula, working quickly but gently. The cap was replaced immediately after the breaking of the chunks. More sodium sulfate was added and manually mixed as necessary to produce free-flowing, finely divided slurry. The samples were extracted by rotating end-over-end for at least 30 minutes. Care was be taken to release pressure by opening and closing the flasks at intervals.

The solids were allowed to settle to settle for one to two minutes. The solvent layer was filtered through a small glass funnel containing a layer of anhydrous sodium sulfate over a plug of glass wool into a receiving conical flask. The sodium sulfate was thoroughly pre-wetted with acetone. The sodium sulfate was rinsed with 2 to 3 mL of acetone as Soon as the surface is exposed. The top of the sodium sulfate layer was not allowed to go dry. The sediment sample was extracted twice more by adding approximately 15 mL of acetone/petroleum spirit mixture to the sample, capping the extraction tube tightly, and shaking vigorously by hand for 2 minutes. All the extracts are combined and poured into the round bottom flask of the rotary evaporator. The round bottom flask of the rotary evaporator is placed in a constant temperature hot water bath so that the concentrator flask is partially, but not completely, immersed. The temperature of the bath was adjusted and the position of the apparatus so that the solvent heat evenly. The sample volume was reduced to approximately 1.0 mL.

### 4.5 Extraction procedure for water

The method employed is EPA method 3510C with slight modifications [9]. 25 mL of dichloromethane was added to 250 mL of water sample (unfiltered) in its original sample bottle. The bottle was closed tightly with an aluminium lined cap. 20 µg/L of internal standard, decafluorobiphenyl was added. The bottle was shaken manually for 15 minutes so that the vortex formed at the surface reaches almost to the bottom of the bottle. The contents of the bottle were transferred to 500 mL separatory funnel. 10 minutes was allowed for the aqueous and the organic phase to separate. The organic layer was transferred to 250 mL separatory funnel and the aqueous layer was returned to the sample bottle. The 500 mL separatory funnel was rinsed twice with dichloromethane; 10 mL at first and then 15 mL, transferring the solvent to the sample bottle after each rinsing.

The shaking, separation and rinsing procedures were repeated twice. After the third separation the organic layer was transferred to the 250 separatory funnel and the sample discarded. The 500 mL separatory funnel was rinsed again and the contents added to the 250 mL separatory funnel. The 250 mL separatory funnel was shaken for 2 minutes and allowed to stand for 10 minutes. 5 cm^3^ of anhydrous sodium sulfate was placed in a 125 mL sintered glass funnel, was set up to drain into a 250 mL round bottom flask. The organic layer in the 250 mL separatory funnel was drained into the filtration column. 15 mL of dichloromethane was added to the aqueous layer remaining in the separatory funnel and shaken for 2 minutes and then allowed to stand for 10 minutes. The organic layer was drained again through the sodium sulfate filter column and the remaining aqueous layer phase discarded. The 250 separatory funnel was rinsed twice with 10 mL of dichloromethane and passed through the sodium sulfate column. The sodium sulfate column was washed with 10 mL of dichloromethane. The column was removed and 1.5 mL of iso-octane was added to the 250 mL round bottom flask as a keeper. The combined sample extracts were evaporated under vacuum using a rotary evaporator at 30-35°C to 5 or 6 mL. The concentrate was transferred with 4×1 mL rinsings to a 15 mL graduated glass tube with conical bottom. The evaporation was finished to 3 mL under gentle stream of nitrogen at 50 to 60°C (water bath) at atmospheric pressure. The extract is solvent exchanged to iso-octane.

### 4.6 Cleanup for water and sediment

A 600 mm x 19 mm i.d. cleanup column was prepared by blocking the hole with glass wool and adding 3g of activated silica gel (60 to 100 mesh, calcined at 650°C for 24 h, and then stored at 130°C until use. The column was topped with 1 cm of preheated Na_2_SO_4_ previously heated at 650°C for 18 h, and stored in a clean bottle in a dessicator. The column was eluted with 40 mL hexane and discarded. The concentrated extract in iso-octane was transferred to the column and eluted with 50 mL of 20 + 80 DCM/hexane (v/v ratio). The eluent was collected in a 100 mL round bottom flask. This fraction (referred as eluent 1) contains PCBs and about 14°C.

The elusion was continued with another 50 mL of 50 + 49.65 + 0.35 DCM/hexane/acetonitrile mixture and the eluate collected in another 100 mL round bottom flask. This fraction called eluate 2 contains endosulfan, dieldrin, endrin, and methoxychlor. The eluates are reduced by volume with rotary evaporator to 3 mL and solvent exchanged to iso-octane and the volume is further reduced to 1 mL in a stream of nitrogen.

## Limitations

Lack of historical data on PCBs and OCPs levels in intertidal sediment and surface-mixed layer water in the lagoon ecosystem made it challenging to evaluate long-term trends and impacts. Besides, limited budget constrained the scope of target micropollutants’ analysis, the length of seasonal campaign, and the number of sampling locations considered in this study.

## Ethics Statement

This study did not involve human or animal subjects, and no data from social media platforms were used.

## CRediT authorship contribution statement

**Nsikak U. Benson:** Conceptualization, Visualization, Investigation, Data curation, Writing – original draft, Writing – review & editing. **John P. Unyimadu:** Conceptualization, Methodology, Investigation, Data curation, Writing – original draft, Writing – review & editing. **Imokhai T. Tenebe:** Conceptualization, Data curation, Writing – original draft, Writing – review & editing.

## Data Availability

Supplementary data for PCBs and OCPs in surface-mixed layer water and sediment from Lagos lagoon (Original data) (Mendeley Data)
